# AFTER REVIEW AND TIPS FROM NIH STAFF

**DOI:** 10.1093/geroni/igae098.1804

**Published:** 2024-12-31

**Authors:** Emerald Nguyen

**Affiliations:** National Institutes of Health, Bethesda, Maryland, United States

## Abstract

After the review meeting, the SRO prepares the summary statement, which is the primary outcome of peer review. Program staff and advisory councils at the institutes use this official document to make recommendations about which applications to fund. How can you best prepare for the next steps for your application? Understand the qualities of a competitive NIH application, talk to program staff, and take advantage of the opportunity to become a peer reviewer yourself. From the field, apply the tips shared by applicants and reviewers about what they do and what they look for in a well-written application. Receiving the summary statement can be daunting and discouraging; but by de-mystifying the process and getting involved, you can help position your application towards maximum success.

